# Incorporating uric acid into the CHA2DS2-VASc score improves the prediction of new-onset atrial fibrillation in patients with acute myocardial infarction

**DOI:** 10.1186/s12872-023-03561-9

**Published:** 2023-10-27

**Authors:** Xuefeng Wu, Yi Zhang, Xili Yang, Zhaoyan Xu, Yingqing Feng

**Affiliations:** 1 The Second School of Clinical Medicine, Southern Medical University, Guangzhou, China; 2 Department of Cardiology, The First People Hospital of Foshan, Foshan, China; 3 Department of Cardiology, Hypertension Research Laboratory, Guangdong Cardiovascular Institute, Guangdong Provincial People’s Hospital, Guangdong Academy of Medical Sciences, Southern Medical University, No. 106, Zhongshan 2nd Road, Yuexiu District, Guangzhou, 510080 China

**Keywords:** New-onset atrial fibrillation, Acute Myocardial Infarction, CHA2DS2-VASc score, Uric acid

## Abstract

**Background:**

New-onset atrial fibrillation (NOAF) is a common cardiac arrhythmia observed in patients with acute myocardial infarction (AMI) and is associated with worse outcomes. While uric acid has been proposed as a potential biomarker for predicting atrial fibrillation, its association with NOAF in patients with AMI and its incremental discriminative ability when added to the CHA2DS2-VASc score are not well established.

**Methods:**

We conducted a retrospective analysis of 1000 consecutive patients with AMI without a history of atrial fibrillation between January 2018 and December 2020. Continuous electrocardiographic monitoring was performed during the patients’ hospital stay to detect NOAF. We assessed the predictive ability of the different scoring models using receiver operating characteristic (ROC) curves. In addition, we employed the area under the curve (AUC), integrated discrimination improvement (IDI), and net reclassification improvement (NRI) analyses to assess the incremental discriminative ability of uric acid when added to the CHA2DS2-VASc score.

**Results:**

Ninety-three patients (9.3%) developed NOAF during hospitalisation. In multivariate regression analyses, the adjusted odds ratio (OR) for NOAF was 1.439 per one standard deviation increase in uric acid level (95% confidence intervals (CI):1.182–1.753, p < 0.001). The ROC curve analysis revealed that the AUC for uric acid was 0.667 (95% CI:0.601–0.719), while the AUC for the CHA2DS2-VASc score was 0.678 (95% CI:0.623–0.734). After integrating the uric acid variable into the CHA2DS2-VASc score, the combined score yielded an improved AUC of 0.737 (95% CI:0.709–0.764, p = 0.009). Furthermore, there was a significant improvement in both IDI and NRI, indicating an incremental improvement in discriminative ability (IDI = 0.041, p < 0.001; NRI = 0.627, p < 0.001).

**Conclusion:**

Our study suggests that uric acid level is an independent risk factor for the development of NOAF after AMI. Furthermore, the incorporation of uric acid into the CHA2DS2-VASc score significantly improves the discriminative ability of the score in identifying patients at high risk for NOAF.

**Supplementary Information:**

The online version contains supplementary material available at 10.1186/s12872-023-03561-9.

## Introduction

Atrial fibrillation is a prevalent arrhythmia in patients with acute myocardial infarction (AMI) with reported incidence rates ranging from 6–21% [1]. Studies have shown that patients with AMI who develop atrial fibrillation experience significantly increased mortality and hospitalisation rates for heart failure compared to those with sinus rhythm [2, 3]. According to the sequence of occurrence, atrial fibrillation can be classified into two types: preexisting atrial fibrillation prior to AMI and new-onset atrial fibrillation (NOAF) after AMI. Research has indicated that NOAF after AMI carries a higher risk of ischaemic stroke and mortality than pre-existing atrial fibrillation [4, 5]. Therefore, identifying high-risk individuals prone to developing NOAF after AMI is crucial.

The CHA2DS2-VASc score, recommended by the guidelines for assessing the risk of embolism in patients with atrial fibrillation [6], is increasingly utilised to predict the risk of atrial fibrillation development [7, 8]. However, its predictive ability for NOAF in patients with AMI is considered unsatisfactory [9, 10]. In recent years, there has been growing interest in the relationship between uric acid and atrial fibrillation. Studies have indicated that uric acid may play a role in the onset and progression of atrial fibrillation through mechanisms such as oxidative stress, inflammation, fibrosis, and cell apoptosis [11]. Population-based and clinical evidence have demonstrated that hyperuricaemia significantly increases the risk of developing atrial fibrillation [12–14]. However, most studies conducted thus far have focused on the general population or individuals with multiple high-risk factors for cardiovascular diseases, with limited research available on the correlation between uric acid and NOAF, specifically in patients with AMI. Therefore, in this study, we aimed to assess the relationship between uric acid levels and the emergence of NOAF in patients with AMI during hospitalisation. Additionally, we aimed to evaluate whether the inclusion of uric acid as an indicator could enhance the predictive capacity of the CHA2DS2-VASc score.

## Methods

### Study participants

In the present study, an initial number of 1030 consecutive patients diagnosed with AMI who underwent percutaneous coronary intervention at the First People Hospital of Foshan from January 2018 to December 2020 were retrospectively enrolled. After excluding one patient with a history of prior mitral valve replacement, two patients with a history of atrial fibrillation, and 27 patients without uric acid data, the current study population consisted of 1000 patients. This study was approved by the Ethics Committee of the First People Hospital of Foshan (2019-05-25). The requirement for informed consent was waived by the Ethics Committee of the First People Hospital of Foshan because of its retrospective nature. The study adhered to the Declaration of Helsinki, and all procedures were in accordance with the approved guidelines.

### Definition of diagnoses

AMI was defined as evidence of myocardial injury based on elevated cardiac troponin levels along with at least one of the following criteria: myocardial ischaemia symptoms, ischaemic electrocardiogram changes, the presence of a pathological Q wave on the electrocardiogram, or echocardiographic evidence of a new regional wall motion abnormality. The ST-segment elevation myocardial infarction (STEMI) and non-ST-segment elevation myocardial infarction (NSTEMI) subtypes of AMI were further categorized using the fourth universal definition of myocardial infarction [15].

In this study, all patients underwent continuous electrocardiography monitoring to identify arrhythmias during their hospital stay in either the cardiac care unit or the regular cardiac ward. Any newly diagnosed atrial fibrillation that appeared during hospitalisation, characterised by the absence of P-waves, atrial activity represented by fibrillatory waves, and irregular RR intervals lasting at least 30 s, was referred to as NOAF. Additionally, atrial flutter was considered equivalent to atrial fibrillation.

### Data collection

The electronic medical records were searched for clinical and laboratory information. Blood samples were drawn from the patients’ veins the morning after admission and analysed for biochemical variables using a SIEMENS ADVIA Chemistry XPT automatic analyser. The estimated glomerular filtration rate (eGFR) was calculated using the Chinese version of the Modification of Diet in Renal Disease (MDRD) formula [16]. Chronic kidney disease (CKD) was defined as an eGFR < 60 ml/min/1.73 m^2^.

Echocardiographic examinations were performed within 12–24 h of hospitalisation. The left atrial diameter (LAD) was determined from M-mode echocardiographic images using a leading‐edge‐to‐leading‐edge method, measuring the maximal distance between the posterior aortic root wall and the posterior left atrial wall at end‐systole. The left ventricular ejection fraction (LVEF) was assessed using Simpson’s method, whereby it was calculated by subtracting the end-systolic left ventricular volume from the end-diastolic left ventricular volume and subsequently dividing the result by the end-diastolic left ventricular volume. Left atrial enlargement (LAE) was defined as LAD ≥ 3.9 cm in women and LAD ≥ 4.1 cm in men [17].

Coronary angiography and intervention therapy were performed by experienced physicians based on current guidelines and clinical practices. Coronary artery severity was assessed using the SYNTAX score [18] using the online calculator version available at www.syntaxscore.com.

### Risk score calculation

To determine the CHA2DS2-VASc score, one point was assigned for each of the following factors: congestive heart failure, hypertension, age between 65 and 74 years, diabetes mellitus, vascular disease, and female sex. Additionally, two points were given to patients aged 75 years or older and a history of stroke or transient ischaemic attack.

### Statistical analysis

Dichotomous variables were expressed as percentages and analysed using either the chi-square test or Fisher’s exact test. To assess normal distribution, the Kolmogorov-Smirnov test was employed for continuous variables. For normally distributed data, the mean and standard deviation were calculated and analysed using the Student’s t-test. For non-normally distributed variables, the median and interquartile range were reported, and the Mann-Whitney U test was used for analysis.

We used binary logistic regression models to assess the association between NOAF and a 1 standard deviation (SD) increase in uric acid levels and estimated the corresponding odds ratios (OR) and 95% confidence intervals (CI). We also categorised uric acid levels into tertiles and used the lowest tertile as the reference category to determine the OR and CI for each tertile. To mitigate potential confounding effects, adjustments were made for variables with a p-value of less than 0.05 in the univariate logistic analyses. To confirm the validity of our findings, we performed a sensitivity analysis in patients who did not have a history of hyperuricaemia, as the use of uric acid-lowering drugs could potentially affect the level of uric acid at admission. We also conducted an additional sensitivity analysis in patients without CKD because renal function is known to have a significant influence on uric acid levels. Considering that equating atrial flutter with atrial fibrillation may introduce bias, we performed a sensitivity analysis after excluding atrial flutter and considering only atrial fibrillation as NOAF.

A receiver operating characteristic (ROC) curve was plotted to evaluate the discriminatory ability of the CHA2DS2-VASc score, and the corresponding area under the curve (AUC) was calculated. Furthermore, net reclassification index (NRI) and integrated discrimination improvement (IDI) methods were used to determine whether the inclusion of uric acid to the CHA2DS2-VASc score improved its predictive capability.

Survival curves were estimated using the Kaplan–Meier (KM) method and compared using the log-rank test. To determine the independent predictors of in-hospital mortality, multivariate Cox proportional-hazards models were used to calculate the hazard ratios (HR) and 95% CI with adjustment for covariates with a p-value of less than 0.05 in the univariate Cox regression analyses. Statistical analyses were conducted using SPSS (version 26.0, SPSS Inc., Chicago, Illinois, USA), MedCalc version 19.1 (MedCalc Software, Belgium), and the R Programming Language (version 4.2.1). Statistical significance was considered when the two-tailed p-value was less than 0.05.

## Results

### Baseline characteristics of the study participants

The baseline characteristics of the study population are shown in Table 1. A total of 1000 patients were included in this study, of whom 93 experienced NOAF during hospitalisation, resulting in a prevalence of 9.3%. The median time from admission to the onset of NOAF was 4 (3,6) days. Compared to the non-NOAF group, patients with NOAF were older and had a higher prevalence of prior stroke, as well as a greater incidence of comorbidities such as CKD. Furthermore, patients with NOAF had significantly higher KILLIP grades and resting heart rates at admission. Laboratory findings revealed that patients with NOAF had lower triglyceride levels but higher levels of uric acid, serum creatine, and N-terminal pro-brain natriuretic peptide (NT-proBNP). Echocardiographic parameters indicated that patients with NOAF had a larger LAD and lower LVEF, while the SYNTAX and CHA2DS2-VASc scores were significantly higher in the NOAF group.

**Table 1:** baseline characteristics of the study population

Variables	Overall (n = 1000)	NOAF (n = 93)	Non-NOAF (n = 907)	p value
Age, years	61.14 ± 12.41	68.63 ± 11.51	60.37 ± 12.25	< 0.001
Male, n(%)	823(82.3)	72(77.4)	751(82.8)	0.195
Smoking, n(%)	349(34.9)	28(30.1)	321(35.4)	0.309
Drinking, n(%)	237(23.7)	23(24.7)	214(23.6)	0.806
STEMI, n(%)	556(55.6)	48(51.6)	508(56.0)	0.416
HT, n(%)	495(49.5)	50(53.8)	445(49.1)	0.388
DM, n(%)	245(24.5)	25(26.9)	220(24.3)	0.575
Hyperlipidemia, n(%)	390(39.0)	36(38.7)	354(39.0)	0.952
History of hyperuricemia, n(%)	194(19.4)	20(21.5)	174(19.2)	0.590
History of stroke, n(%)	67(6.7)	11(11.8)	56(6.2)	0.038
History of COPD, n(%) History of HF, n(%)	13(1.3) 92(9.2)	1(1.1) 11(11.8)	12(1.3) 81(8.9)	1.000 0.357
History of MI, n(%)	12(1.2)	1(1.1)	11(1.2)	1.000
History of PCI, n(%)	50(5.0)	7(7.5)	43(4.7)	0.217
PAD, n(%)	9(0.9)	2(2.2)	7(0.8)	0.201
CKD, n(%)	128(12.8)	29(31.2)	99(10.9)	< 0.001
KILLIP > 1, n(%)	398(39.8)	60(64.5)	338(37.3)	< 0.001
SBP, mmHg	130.09 ± 21.91	126.96 ± 23.24	130.41 ± 21.76	0.148
DBP, mmHg	79.57 ± 29.15	75.81 ± 14.43	79.96 ± 30.24	0.191
HR at admission, bpm Hb, g/L	79.07 ± 15.16 125.3 ± 42.9	83.53 ± 20.39 119.4 ± 45.4	78.61 ± 14.45 125.9 ± 42.6	0.003 0.171
HbA1c, %	7.03 ± 1.97	7.07 ± 1.89	7.03 ± 1.98	0.877
TC, mmol/L	4.66(4.00-5.42)	4.61(4.00-5.22)	4.68(4.00-5.44)	0.178
LDL-C, mmol/L	2.95(2.37–3.57)	2.92(2.30–3.64)	2.94(2.38–3.57)	0.622
HDL-C, mmol/L	1.01(0.90–1.17)	0.98(0.89–1.15)	1.02(0.90–1.17)	0.272
TG, mmol/L	1.52(1.14–2.08)	1.39(1.05–1.81)	1.53(1.14–2.12)	0.020
UA, µmol/L	374.5(313.0-450.0)	427.0(357.5-529.5)	369.0(309.0-444.0)	< 0.001
NT-proBNP, ng/L	722.0(233.6-1965.3)	2324.0(616.1-6096.5)	722.0(222.0-1731.0)	< 0.001
Scr, µmol/L	76.0(65.0–91.0)	84.0(73.0-115.5)	75.0(64.4–90.0)	< 0.001
eGFR, ml/min/1.73m^2^	92.67 ± 30.08	74.0 ± 31.25	94.58 ± 29.31	< 0.001
LAD, mm	36.0(33.0–38.0)	38.0(34.0–42.0)	36.0(33.0–38.0)	< 0.001
LVEF, % LAE, n(%)	55.5(47.0–62.0) 241(24.1)	48.0(42.0–58.0) 42(45.2)	56.0(48.0–62.0) 199(21.9)	< 0.001 < 0.001
SYNTAX score	14.0(9.0–19.0)	16.0(10.5–22.0)	14.0(9.0–19.0)	0.016
CHA2DS2-VASc score Hospitalization, day Time of NOAF onset, day	2(1–3) 7.0(6.0–10.0) /	3(2–4) 9.0(7.0–13.0) 4(3–6)	2(1–3) 7.0(6.0–10.0) /	< 0.001 < 0.001 /
In hospital mortality, n(%) Medication at discharge Aspirin, n(%)	14(1.4) 955(95.5)	3(3.2) 74(79.6)	11(1.2) 881(97.1)	0.134 < 0.001
P2Y12 receptor inhibitor, n(%)	993(99.3)	92(98.9)	901(99.3)	0.496
Statins, n(%)	962(96.2)	85(91.4)	877(96.7)	0.019
Beta-blocker, n(%)	713(71.3)	59(63.4)	654(72.1)	0.079
ACEI/ARB, n(%)	490(49.0)	36(38.7)	454(50.1)	0.037
Diuretic, n(%)	234(23.4)	41(44.1)	193(21.3)	< 0.001
OAC, n(%)	25(2.5)	17(18.3)	8(0.9)	< 0.001

STEMI, ST-segment elevation myocardial infarction; HT, hypertension; DM, diabetes mellitus; COPD, chronic obstructive pneumonia disease; HF, heart failure; MI, myocardial infarction; PCI, percutaneous coronary intervention; PAD, peripheral artery disease; CKD, chronic kidney disease; SBP, systolic blood pressure; DBP, diastolic blood pressure; HR, heart rate; Hb, hemoglobin; HbA1c, glycosylated hemoglobin; TC, total cholesterol; LDL-C, low-density lipoprotein cholesterol; HDL-C, high-density lipoprotein cholesterol; TG, triglyceride; UA, uric acid; NT-proBNP, N-terminal pro brain natriuretic peptide; Scr, serum creatine; eGFR, estimated glomerular filtration rate; LAD, left atrium diameter; LVEF, left ventricular ejection fraction; LAE, left atrial enlargement; ACEI, angiotensin-converting enzyme inhibitor; ARB, angiotensin receptor blocker, OAC, oral anticoagulant

The average length of hospitalisation in patients with NOAF was significantly longer than that in patients without NOAF. Furthermore, there was a trend towards higher in-hospital mortality in the NOAF group, although the difference between the two groups was not statistically significant. In terms of discharge medications, patients with NOAF had significantly lower prescription rates of aspirin, statins, and angiotensin-converting enzyme inhibitors (ACEI)/angiotensin receptor blockers (ARB), whereas diuretics and oral anticoagulants (OAC) were prescribed more frequently in the NOAF group.

### Relationship between uric acid and NOAF

Table 2 shows the relationship between the uric acid levels and NOAF risk. When uric acid was analysed as continuous data, following adjustments for age, KILLIP > 1, eGFR, diastolic blood pressure, triglycerides, NT-proBNP, LAD, LVEF, SYNTAX score, and heart rate at admission, the OR for NOAF was 1.439 per 1 SD increase (95% CI:1.182–1.753, p < 0.001). This association remained consistent when uric acid values were categorised into tertiles. In the fully adjusted model, an elevated risk of NOAF was observed across the uric acid tertiles. Specifically, the adjusted OR and 95% CI for NOAF in the third tertile of uric acid compared with the corresponding first tertile was 2.954 (95% CI:1.632–5.348, p < 0.001).

**Table 2:** the relationship between uric acid and NOAF

	Crude OR (95% CI)	p value	Model 1 OR (95% CI)	p value	Model 2 OR (95% CI)	p value
UA (Per 1 SD increase)	1.579(1.306–1.909)	< 0.001	1.590(1.308–1.934)	< 0.001	1.439(1.182–1.753)	< 0.001
Tertile of UA						
T1	reference		reference		reference	
T2	1.359(0.723–2.554)	0.340	1.383(0.729–2.623)	0.321	1.455(0.753–2.813)	0.265
T3	3.186(1.818–5.583)	< 0.001	3.412(1.926–6.046)	< 0.001	2.954(1.632–5.348)	< 0.001
p for trend		< 0.001		< 0.001		< 0.001

Model 1: adjusted for age and sex

Model 2: adjusted for age, KILLIP > 1, eGFR, diastolic blood pressure, TG, NT-proBNP, LAD, LVEF, SYNTAX score, and heart rate at admission

To evaluate the robustness of the association between uric acid and NOAF, we performed a series of sensitivity analyses. The results of these analyses are shown in Supplementary Table 1. Following adjustments for confounding factors, the association between uric acid and NOAF remained consistent in the subgroups of patients without CKD and those without a history of hyperuricaemia, in line with the overall population. During hospitalisation, six cases of atrial flutter events were documented. After excluding atrial flutter and focusing solely on atrial fibrillation as NOAF, the independent predictive value of uric acid persisted.

### The additive effect of uric acid on the CHA2DS2-VASc score

**Fig. 1:**
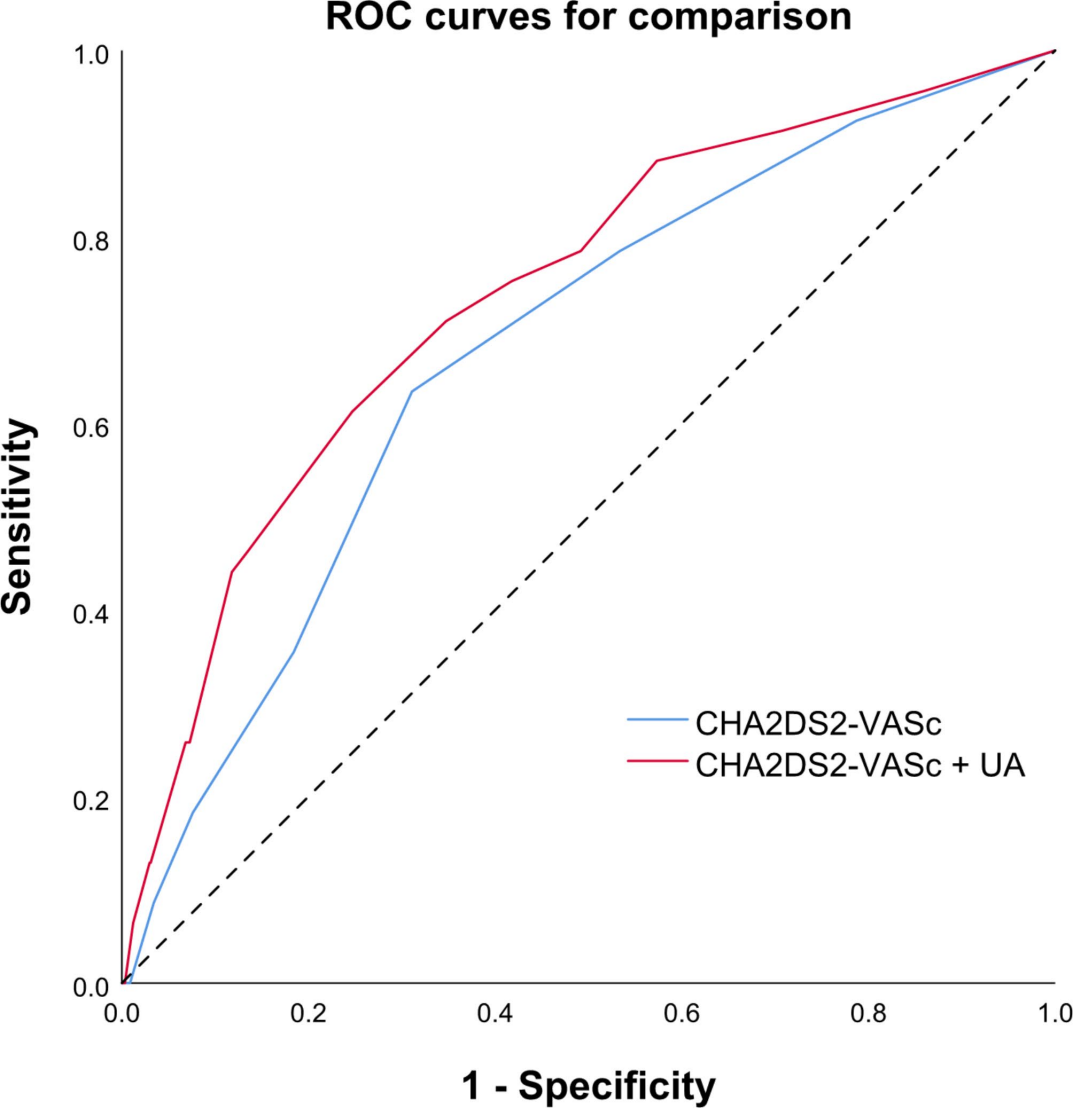
Receiver operating characteristic curves analysis for CHA2DS2-VASc score and CHA2DS2-VASc combined with UA in predicting NOAF

We performed ROC analysis to evaluate the diagnostic utility of uric acid and CHA2DS2-VASc scores in identifying patients with NOAF. The AUC for uric acid and CHA2DS2-VASc score were 0.667 (95%CI:0.601–0.719) and 0.678 (95%CI:0.623–0.734), respectively. We applied the ROC curve-derived optimal cutoff value to convert uric acid level into a binary variable and subsequently integrated this binary uric acid variable into the CHA2DS2-VASc score. The combined score yielded an AUC of 0.737 (95% CI:0.709–0.764), which was statistically significantly greater than the AUC of the CHA2DS2-VASc score alone (Fig. 1).

To further evaluate the additional discriminative ability of uric acid, we assessed the IDI and NRI. Our findings showed a significant incremental improvement in both the IDI and NRI when uric acid was incorporated into the CHA2DS2-VASc score compared with utilising the CHA2DS2-VASc score alone (Table 3).

**Table 3:** comparison of AUC, IDI, and NRI between CHA2DS2-VASc and its combination with UA

	AUC (95% CI)	p value	IDI (95% CI)	p value	NRI (95% CI)	p value
CHA2DS2-VASC	0.678(0.648–0.707)		Ref		Ref	
CHA2DS2-VASC + UA	0.737(0.709–0.764)	0.009	0.041(0.027–0.055)	< 0.001	0.627(0.428–0.825)	< 0.001

### Relationship between uric acid and in-hospital mortality

**Fig. 2:**
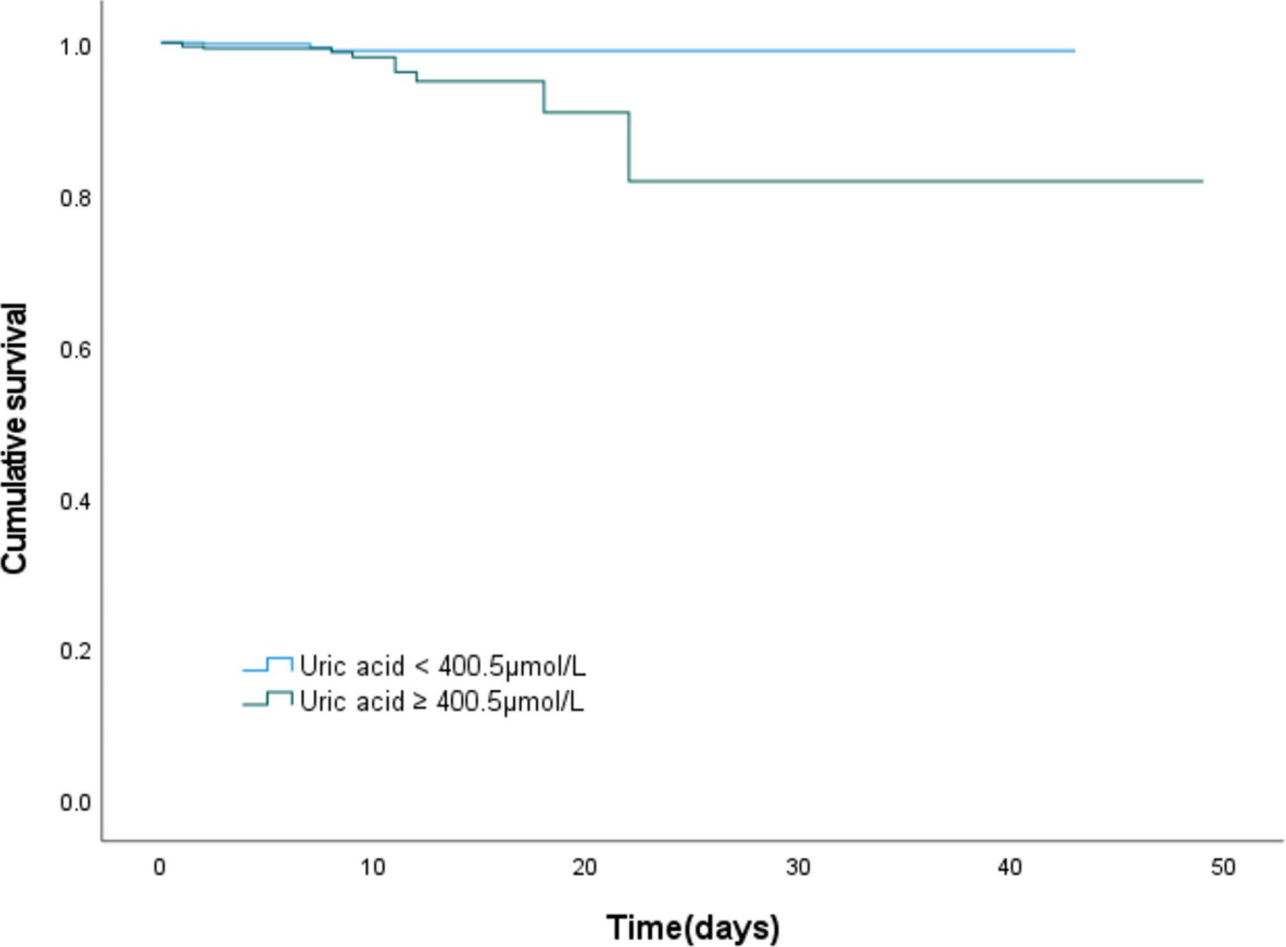
Kaplan-Meier Curve of survival stratified by uric acid level, with log-rank test = 0.029

Through multivariate Cox regression analyses, after adjustments for covariates, including hypertension, chronic obstructive pulmonary disease (COPD), eGFR, uric acid, history of heart failure (HF), uric acid was found to be an independent predictor of in-hospital mortality (Supplementary Table 2). Using the cutoff value established by the ROC curve of uric acid, patients with higher uric acid levels displayed an increased incidence of in-hospital mortality (Fig. 2). However, we did not observe any difference in in-hospital mortality between patients with and without NOAF.

## Discussion

The findings of this study revealed that the uric acid level was an independent predictor of NOAF during hospitalisation in patients diagnosed with AMI. Furthermore, incorporating the uric acid indicator into the CHA2DS2-VASc score resulted in a significant improvement in the discriminatory performance of the score in identifying patients at a higher risk of developing NOAF.

NOAF is a common cardiac arrhythmia in patients diagnosed with AMI. The incidence rate of NOAF in this study was 9.3%, which is consistent with previous research [1]. Studies have indicated that patients with AMI and NOAF have markedly increased mortality rates and hospitalization rates for heart failure compared to those with sinus rhythm [2, 3]. Consistently, in this study, patients who experienced NOAF had prolonged hospital stays, and although the difference was not statistically significant, they exhibited a higher in-hospital mortality rate than patients without NOAF. The in-hospital mortality rate reported in this study was significantly lower than the one reported in the China Acute Myocardial Infarction Registry [19]. However, it should be considered that the study population was different, and all participants received percutaneous artery intervention, which may have impacted the study outcomes. Additionally, another study with a similar participant profile reported in-hospital mortality rates comparable to those in our study [20].

Recently, the correlation between uric acid and atrial fibrillation has received increasing attention and has been widely studied in different populations. For instance, the AMORIS cohort study conducted in Sweden, which followed 339,604 individuals without cardiovascular risk factors or diseases for an average of 25 years, demonstrated that an elevated baseline uric acid level was significantly associated with an increased risk of atrial fibrillation [12]. A meta-analysis revealed that the association between uric acid and atrial fibrillation was consistent in multiple and diverse populations [21]. Furthermore, Mantovani et al. reported that hyperuricaemia is an independent risk factor for atrial fibrillation in patients with type 2 diabetes [13], while another study revealed a significant correlation between uric acid levels and the incidence of atrial fibrillation in patients with hypertension [14]. In patients who undergo radiofrequency ablation, uric acid levels are closely related to postoperative recurrence [22]. Moreover, several studies have explored the dose-response relationship between uric acid and atrial fibrillation, with findings indicating that each 1 mg/dL increase in serum uric acid levels was associated with a 15% increase in atrial fibrillation risk in men and a 35% increase in women [23]. Similarly, another study identified a dose-response relationship between uric acid levels and atrial fibrillation risk, with higher quartiles of uric acid levels exhibiting an increased risk of atrial fibrillation compared with the lowest quartile. The adjusted risk ratios were 1.09 (95% CI:1.06–1.12), 1.19 (95% CI:1.16–1.23), and 1.45 (95% CI:1.41–1.49) for the second, third, and fourth quartiles, respectively [12]. In the present study, even after adjusting for traditional atrial fibrillation risk factors, the uric acid level remained an independent predictor of atrial fibrillation, further confirming the correlation between the uric acid level and atrial fibrillation in patients with AMI. Additionally, in the multivariate analysis of this study, the uric acid level showed a dose-response relationship with an increased risk of atrial fibrillation both as a categorical and continuous variable. These findings are consistent with those of previous studies. Previous research has shown that the association between uric acid and atrial fibrillation risk may differ by sex, with a stronger correlation observed in women [24]. However, in the current study, as the majority of patients were male and the sample size was limited, it remains uncertain whether there is a sex difference in the relationship between uric acid levels and the risk of NOAF after AMI. Further research is warranted to elucidate whether such sex-based differences exist.

The relationship between uric acid and atrial fibrillation is a topic of ongoing research and debate. While many studies have found a correlation between high uric acid levels and atrial fibrillation, there is also a significant overlap between hyperuricaemia and other atrial fibrillation risk factors and comorbidities, such as hypertension, metabolic syndrome, and diabetes [25], and there is a potential interaction between uric acid and insulin resistance that could contribute to an elevation in arterial stiffness [26]. Despite this, Mendelian randomisation analysis has provided evidence of a causal genetic relationship between uric acid levels and atrial fibrillation risk [27]. Uric acid is a compound involved in human purine metabolism and has been shown to have both antioxidant and pro-oxidant properties, depending on its concentration [28]. Additionally, uric acid is closely associated with cytokines such as C-reactive protein and Interleukin-6 [29]. Some studies have proposed that it can promote development of atrial fibrillation by triggering oxidative stress and inflammation [30, 31]. Although a meta-analysis showed the potential benefit of hyperuricaemia treatment in patients with atrial fibrillation [32], conclusive evidence is lacking. Clinical trials based on the type or origin of atrial fibrillation could help to better understand the role of urate-lowering drugs [33]. Overall, although the precise mechanisms underlying the relationship between uric acid and atrial fibrillation remain unclear, it is clear that uric acid levels are an important consideration in assessing atrial fibrillation risk.

We observed that patients who developed NOAF exhibited both a larger LAD and a higher proportion of LAE, consistent with the findings of a previous observational study [34]. Available evidence indicates that LAE serves as a marker of increased risk of atrial fibrillation [35]. Data from the Framingham Heart Study demonstrated that an estimated 24% association between alcohol consumption and atrial fibrillation risk could be explained by LAE [36]. Given these insights, it is plausible to speculate that the association between uric acid and atrial fibrillation be partly attributed to the presence of LAE. However, even in a subgroup of patients with LAE, an association between elevated uric acid levels and a heightened likelihood of atrial fibrillation remains [14]. This highlights the importance of uric acid management in patients with LAE as a strategy to mitigate the risk of atrial fibrillation.

The CHA2DS2-VASc score is recommended by the guidelines for managing and stratifying the risk of stroke in patients with atrial fibrillation [6]. However, this score is increasingly used to predict the risk of atrial fibrillation. Several studies have shown that the CHA2DS2-VASc score exhibits good predictive value in various populations, including the general population, patients with heart failure, and those with atrial fibrillation after cardioversion [7, 8, 37]. Nevertheless, its predictive ability for atrial fibrillation after AMI is limited, with studies reporting a low area under the ROC curve for the CHA2DS2-VASc [9, 10]. The results of this study indicate that the area under the ROC curve for the CHA2DS2-VASc in predicting NOAF was 0.678, which is similar to the results of the aforementioned studies. These findings suggest that a more accurate scoring model is required to predict the occurrence of NOAF after AMI.

The current study integrated uric acid with the conventional CHA2DS2-VASc score and demonstrated that the combined model significantly enhanced the area under the ROC curve, IDI, and NRI for predicting atrial fibrillation, as compared with the CHA2DS2-VASc score alone. These findings highlight the potential utility of incorporating uric acid levels into risk stratification models for atrial fibrillation in patients with AMI. Further research is warranted to validate these findings and explore the clinical implications of incorporating uric acid as a risk factor in atrial fibrillation management strategies.

### Limitations

This study has several limitations. First, as this was a single-centre, cross-sectional study, the results need to be confirmed by more multicentre studies. Second, we excluded patients with a history of atrial fibrillation; however, because a considerable proportion of atrial fibrillation patients are asymptomatic [38], there may have been asymptomatic atrial fibrillation patients in the study population who were misclassified as having NOAF. Third, we lacked information on the medication history of the patients prior to admission, and some patients may have used uric acid-lowering drugs or other medications that could have affected the uric acid levels measured after admission. However, we conducted a sensitivity analysis by excluding patients with a history of hyperuricaemia who had previously received uric acid-lowering therapy. The results of this analysis demonstrated that uric acid remained an independent predictor of atrial fibrillation in the remaining population, which is consistent with the results of the overall population. Nevertheless, further research with larger sample sizes and comprehensive medication history data is required to validate these findings and address potential confounding factors. Finally, we primarily focused on NOAF during hospitalisation. Information regarding the evolution of patients with NOAF during the follow-up period is lacking. On the other hand, uric acid levels are subject to various influences, such as lifestyle modifications and drug usage. Extending the scope of the analysis to include longitudinal data on uric acid levels and recurrent atrial fibrillation episodes could yield a more comprehensive understanding of how uric acid fluctuations coincide with the persistence of atrial fibrillation over time.

## Conclusions

In conclusion, we found that uric acid level was a significant and independent risk factor for the development of NOAF after AMI. Furthermore, the integration of uric acid with the conventional CHA2DS2-VASc score improved the predictive accuracy in identifying patients at a high risk for NOAF.

### Electronic supplementary material

Below is the link to the electronic supplementary material.

Supplementary Material 1

## Data Availability

The data are shared on request. Please contact the corresponding author directly to request for data sharing.

## Abbreviations

NOAF: New-onset atrial fibrillation
AMI: Acute myocardial infarction
ROC: Receiver operating characteristic
IDI: Integrated discrimination improvement
NRI: Net reclassification improvement
AUC: Area under the curve
STEMI: ST-segment elevation myocardial infarction
NSTEMI: Non-ST-segment elevation myocardial infarction
CKD: Chronic kidney disease
eGFR: Estimated glomerular filtration rate
MDRD: Modification of Diet in Renal Disease
LAD: Left atrial diameter
LVEF: Left ventricular ejection fraction
LAE: Left atrial enlargement
SD: Standard deviation
OR: Odds ratios
CI: Confidence intervals
NT-proBNP: N-terminal pro brain natriuretic peptide
COPD: Chronic obstructive pulmonary disease
HF: Heart failure
ACEI: Angiotensin-converting enzyme inhibitor
ARB: Angiotensin receptor blocker
OAC: Oral anticoagulant

## References

[1] Schmitt J, Duray G, Gersh BJ, Hohnloser SH (2009). Atrial fibrillation in acute Myocardial Infarction: a systematic review of the incidence, clinical features and prognostic implications. Eur Heart J.

[2] Luo J, Xu S, Li H, Gong M, Li Z, Liu B, Qin X, Shi B, Wei Y (2021). Long-term impact of the burden of new-onset atrial fibrillation in patients with acute Myocardial Infarction: results from the NOAFCAMI-SH registry. Europace.

[3] Hofer F, Kazem N, Hammer A, El-Hamid F, Koller L, Niessner A, Sulzgruber P (2021). Long-term prognosis of de novo atrial fibrillation during acute Myocardial Infarction: the impact of anti-thrombotic treatment strategies. Eur Heart J Cardiovasc Pharmacother.

[4] Fauchier L, Bisson A, Bodin A, Herbert J, Angoulvant D, Danchin N, Cottin Y (2021). Outcomes in patients with acute Myocardial Infarction and new atrial fibrillation: a nationwide analysis. Clin Res Cardiol.

[5] Obayashi Y, Shiomi H, Morimoto T, Tamaki Y, Inoko M, Yamamoto K, Takeji Y, Tada T, Nagao K, Yamaji K (2021). Newly diagnosed Atrial Fibrillation in Acute Myocardial Infarction. J Am Heart Assoc.

[6] Hindricks G, Potpara T, Dagres N, Arbelo E, Bax JJ, Blomström-Lundqvist C, Boriani G, Castella M, Dan GA, Dilaveris PE (2021). : 2020 ESC guidelines for the diagnosis and management of atrial fibrillation developed in collaboration with the European Association for Cardio-Thoracic Surgery (EACTS): the Task Force for the diagnosis and management of atrial fibrillation of the European Society of Cardiology (ESC) developed with the special contribution of the European Heart Rhythm Association (EHRA) of the ESC. Eur Heart J.

[7] Saliba W, Gronich N, Barnett-Griness O, Rennert G (2016). Usefulness of CHADS2 and CHA2DS2-VASc scores in the prediction of New-Onset Atrial Fibrillation: a Population-based study. Am J Med.

[8] Wu Y, Xie Z, Liang W, Xue R, Wu Z, Wu D, He J, Zhu W, Liu C (2021). Usefulness of CHADS2, R2CHADS2, and CHA2DS2-VASc scores for predicting incident atrial fibrillation in Heart Failure with preserved ejection fraction patients. ESC Heart Fail.

[9] Luo J, Dai L, Li J, Zhao J, Li Z, Qin X, Li H, Liu B, Wei Y (2018). Risk evaluation of new-onset atrial fibrillation complicating ST-segment elevation Myocardial Infarction: a comparison between GRACE and CHA(2)DS(2)-VASc scores. Clin Interv Aging.

[10] Fu Y, Pan Y, Gao Y, Yang X, Chen M (2021). Predictive value of CHA2DS2-VASc score combined with hs-CRP for new-onset atrial fibrillation in elderly patients with acute Myocardial Infarction. BMC Cardiovasc Disord.

[11] Deng Y, Liu F, Yang X, Xia Y (2021). The key role of uric acid in oxidative stress, inflammation, fibrosis, apoptosis, and immunity in the pathogenesis of Atrial Fibrillation. Front Cardiovasc Med.

[12] Ding M, Viet NN, Gigante B, Lind V, Hammar N, Modig K (2023). Elevated uric acid is Associated with New-Onset Atrial Fibrillation: results from the Swedish AMORIS cohort. J Am Heart Assoc.

[13] Mantovani A, Rigolon R, Civettini A, Bolzan B, Morani G, Bonapace S, Dugo C, Zoppini G, Bonora E, Targher G (2018). Hyperuricemia is associated with an increased prevalence of paroxysmal atrial fibrillation in patients with type 2 Diabetes referred for clinically indicated 24-h Holter monitoring. J Endocrinol Invest.

[14] Hidru TH, Tang Y, Liu F, Hui S, Gao R, Li D, Yang X, Xia Y (2020). Does serum uric acid Status Influence the Association between Left Atrium Diameter and Atrial Fibrillation in Hypertension patients?. Front Cardiovasc Med.

[15] Thygesen K, Alpert JS, Jaffe AS, Chaitman BR, Bax JJ, Morrow DA, White HD (2019). Fourth universal definition of Myocardial Infarction (2018). Eur Heart J.

[16] Ma YC, Zuo L, Chen JH, Luo Q, Yu XQ, Li Y, Xu JS, Huang SM, Wang LN, Huang W (2006). Modified glomerular filtration rate estimating equation for Chinese patients with chronic Kidney Disease. J Am Soc Nephrol.

[17] Lang RM, Badano LP, Mor-Avi V, Afilalo J, Armstrong A, Ernande L, Flachskampf FA, Foster E, Goldstein SA, Kuznetsova T (2015). Recommendations for cardiac chamber quantification by echocardiography in adults: an update from the American Society of Echocardiography and the European Association of Cardiovascular Imaging. J Am Soc Echocardiogr.

[18] Sianos G, Morel MA, Kappetein AP, Morice MC, Colombo A, Dawkins K, van den Brand M, Van Dyck N, Russell ME, Mohr FW (2005). The SYNTAX score: an angiographic tool grading the complexity of coronary artery Disease. EuroIntervention.

[19] Xu H, Yang Y, Wang C, Yang J, Li W, Zhang X, Ye Y, Dong Q, Fu R, Sun H (2020). Association of Hospital-Level Differences in Care with outcomes among patients with Acute ST-Segment Elevation Myocardial Infarction in China. JAMA Netw Open.

[20] Li Z, Liu Q, Liu F, Hidru TH, Yang Y, Wang S, Bai L, Chen J, Yang X, Xia Y (2022). Atrial cardiomyopathy markers and new-onset atrial fibrillation risk in patients with acute Myocardial Infarction. Eur J Intern Med.

[21] Tamariz L, Hernandez F, Bush A, Palacio A, Hare JM (2014). Association between serum uric acid and atrial fibrillation: a systematic review and meta-analysis. Heart Rhythm.

[22] Chen Y, Wu Y, Chu X, Wang M (2022). Meta-analysis of the correlation between recurrence of atrial fibrillation and serum uric acid level after radiofrequency ablation. Am J Transl Res.

[23] Xiong J, Shao W, Yu P, Ma J, Liu M, Huang S, Liu X, Mei K (2022). Hyperuricemia is Associated with the risk of Atrial Fibrillation Independent of sex: a dose-response Meta-analysis. Front Cardiovasc Med.

[24] Peters SAE, Woodward M (2019). Established and novel risk factors for atrial fibrillation in women compared with men. Heart.

[25] Feig DI, Kang DH, Johnson RJ (2008). Uric acid and cardiovascular risk. N Engl J Med.

[26] Cassano V, Crescibene D, Hribal ML, Pelaia C, Armentaro G, Magurno M, Toscani A, Miceli S, Andreozzi F, Maio R et al. Uric acid and vascular damage in Essential Hypertension: role of insulin resistance. Nutrients 2020, 12(9). 10.3390/nu12092509 PMC7551393 32825165

[27] Hong M, Park JW, Yang PS, Hwang I, Kim TH, Yu HT, Uhm JS, Joung B, Lee MH, Jee SH (2020). A mendelian randomization analysis: the causal association between serum uric acid and atrial fibrillation. Eur J Clin Invest.

[28] Kang DH, Ha SK (2014). Uric acid puzzle: dual role as anti-oxidantand pro-oxidant. Electrolyte Blood Press.

[29] Zharikov S, Krotova K, Hu H, Baylis C, Johnson RJ, Block ER, Patel J (2008). Uric acid decreases NO production and increases arginase activity in cultured pulmonary artery endothelial cells. Am J Physiol Cell Physiol.

[30] Li P, Zhang L, Zhang M, Zhou C, Lin N (2016). Uric acid enhances PKC-dependent eNOS phosphorylation and mediates cellular ER stress: a mechanism for uric acid-induced endothelial dysfunction. Int J Mol Med.

[31] Li J, Wang S, Bai J, Yang XL, Zhang YL, Che YL, Li HH, Yang YZ (2018). Novel role for the Immunoproteasome Subunit PSMB10 in Angiotensin II-Induced Atrial Fibrillation in mice. Hypertension.

[32] Gao Z, Shi H, Xu W, Guan Z, Su X, Guo N, Ma H. Hyperuricemia Increases the Risk of Atrial Fibrillation: A Systematic Review and Meta-Analysis. *Int J Endocrinol* 2022, 2022:8172639. 10.1155/2022/8172639 PMC9420608 36046801

[33] Yang J, Lou L, Zhang X, Chen Y, Zhou W, Zhang C, Guo X, Hu S (2022). The relationship between Uric Acid and the development, complication, and prognosis of Atrial Fibrillation: the views from a clinical study. Int J Endocrinol.

[34] Chao TF, Hung CL, Chen SJ, Wang KL, Chen TJ, Lin YJ, Chang SL, Lo LW, Hu YF, Tuan TC (2013). The association between hyperuricemia, left atrial size and new-onset atrial fibrillation. Int J Cardiol.

[35] Sardana M, Lessard D, Tsao CW, Parikh NI, Barton BA, Nah G, Thomas RC, Cheng S, Schiller NB, Aragam JR et al. Association of Left Atrial Function Index with Atrial Fibrillation and Cardiovascular Disease: The Framingham Offspring Study. *J Am Heart Assoc* 2018, 7(7). 10.1161/JAHA.117.008435 PMC5907604 29602764

[36] McManus DD, Yin X, Gladstone R, Vittinghoff E, Vasan RS, Larson MG, Benjamin EJ, Marcus GM. Alcohol Consumption, Left Atrial Diameter, and Atrial Fibrillation. J Am Heart Assoc 2016, 5(9). 10.1161/JAHA.116.004060 PMC5079048 27628571

[37] Vitali F, Serenelli M, Airaksinen J, Pavasini R, Tomaszuk-Kazberuk A, Mlodawska E, Jaakkola S, Balla C, Falsetti L, Tarquinio N (2019). CHA2DS2-VASc score predicts atrial fibrillation recurrence after cardioversion: systematic review and individual patient pooled meta-analysis. Clin Cardiol.

[38] Sanna T, Diener HC, Passman RS (2014). Cryptogenic Stroke and atrial fibrillation. N Engl J Med.

