# Supplementary material for: Incorporating uric acid into the CHA2DS2-VASc score improves the prediction of new-onset atrial fibrillation in patients with acute myocardial infarction
Source: BMC Cardiovasc Disord. 2023 Oct 27;23:522. doi: 10.1186/s12872-023-03561-9 (PMC10612166; doi:10.1186/s12872-023-03561-9)
Supplement: Supplementary file 1 — Supplementary Material 1 [file 12872_2023_3561_MOESM1_ESM.docx]

Supplementary Table 1: sensitivity analysis for the association between UA and NOAF

|  |  | Crude  OR (95% CI) | p value | Model 1  OR (95% CI) | p value | Model 2  OR (95% CI) | p value |
| --- | --- | --- | --- | --- | --- | --- | --- |
| UA (per 1 SD increase) | Patients without CKD  (n = 872) | 1.677(1.288-2.183) | < 0.001 | 1.891(1.432-2.498) | < 0.001 | 1.678(1.263-2.229) | < 0.001 |
|  |  |  |  |  |  |  |  |
|  | Patients without a history of hyperuricemia  (n = 806)  Only consider atrial fibrillation as NOAF | 1.777(1.380-2.287)  1.613(1.328-1.959) | < 0.001  < 0.001 | 1.825(1.415-2.353)  1.622(1.329-1.980) | < 0.001  < 0.001 | 1.616(1.251-2.087)  1.474(1.206-1.801) | < 0.001  < 0.001 |

Model 1: adjusted for age and sex.

Model 2: adjusted for age, KILLIP > 1, eGFR, diastolic blood pressure, TG, NT-proBNP, LAD, LVEF, SYNTAX score, and heart rate at admission.

Supplementary Table 2: predictors of in-hospital mortality by Cox proportional hazard regression analysis.

| Variable | HR (95% CI) | p value |
| --- | --- | --- |
| UA | 1.006(1.002-1.010) | <0.001 |
| eGFR | 0.977(0.957-0.998) | 0.031 |
